# Does long sleep duration increase risk of metabolic syndrome in Azar cohort study population?

**DOI:** 10.15171/hpp.2018.41

**Published:** 2018-10-27

**Authors:** Alireza Ostadrahimi, Zeinab Nikniaz, Elnaz Faramarzi, Asghar Mohammadpoorasl, Khalil Ansarin, Mohammad Hossein Somi

**Affiliations:** ^1^Nutrition Research Center, Tabriz University of Medical Sciences, Tabriz, Iran; ^2^Liver and Gastrointestinal Diseases Research Center, Tabriz University of Medical Sciences, Tabriz, Iran; ^3^Department of Statistics and Epidemiology, Tabriz University of Medical Sciences, Tabriz, Iran; ^4^Tuberculosis & Lung Diseases Research Center, Tabriz University of Medical Sciences, Tabriz, Iran

**Keywords:** Sleep duration, Napping, Metabolic syndrome, Hypnotic drugs

## Abstract

**Background:** We decided to assess the correlation between metabolic syndrome (MetS) risks,sleep and napping duration in Azar cohort population according to the increasing incidence of MetS in the world and inconsistence results about sleep duration and MetS.

** Methods:** In this cross-sectional study, MetS and sleep habits of 14916 subjects (35-70 years old) who inhabited in Shabestar city were determined by ATPIII and Pittsburg questionnaire respectively. Inclusion criteria were subjects with 35-70 years old and living in Shabestar for at least 9 months of the year.

**Results:** According to the results, age, living place, body mass index, hypnotic drug use, sleep and napping duration and TV time were the risk factors of MetS. In this regard, long sleep duration (>9 h/24 h), napping (0.25-2 h/day), hypnotic drug use and watching TV (2 h/day)increased the risk of MetS by 1.18 (1.05-1.33), 1.16(1.07-1.26), 1.35(1.13-1.60), and 1.13(1.04-1.23) respectively.

**Conclusion:** According to these results, it appears that proper education for improvement of sleep habit is necessary to reduce incidence of MetS and its consequences. However, there is need for more longitudinal researches and using objective method of sleep habits evaluation for more precise results.

## Introduction

Metabolic syndrome (MetS) includes a set of abnormalities such as hypertension, dyslipidemia, impaired glucose tolerance and abdominal obesity.^1^ According to the results of recent studies, prevalence of MetS along with obesity is increasing in the world especially in developing countries that may be due to the changes in life styles and food habit. It is estimated that about a quarter of the world population has MetS.^2^ Traditionally, obesity and sedation life style are considered as the main contributors of the MetS development.^3^ Nowadays, other new factors such as socio-economic status^4^ and sleep duration,^5^ have been proposed as risk factors in metabolic syndrome development.

Recently , Koren and Taveras noted that sleep deprivation and long sleep duration are associated with increasing the risk of central obesity, metabolic syndrome, cardiovascular diseases and mortality.^5^ While, recent systematic review and meta-analysis studies revealed that the relationship between sleep duration and MetS are inconsistence.^6-8^ In this regard, Xi et al reported no significant relationship between long sleep duration and MetS.^7^ Moreover, Iftikhar et al could not confirm a significant correlation between long sleep duration and metabolic syndrome.^6^ Well designed studies with large sample size are needed to clarify the relationship between sleep duration and MetS on the basis of aforementioned studies.

Considering the increasing incidence of MetS in the world and inconsistent results about the associations between sleep duration and MetS, we decided to assess the correlations between MetS risks, sleep and napping duration in Azar cohort population.

## Materials and Methods

### Participants and procedures

This cross-sectional study was approved by Ethics Committee of Tabriz University of Medical Sciences. We used a part of Azar’s cohort study data in this study.

All eligible individuals from 35 up to 70 years old in Shabestar region were invited to participate in the study from October 2014 to January 2017, and those who were signed the consent form are included in the Azar cohort study. Those included the inhabitants in Shabestar for at least 9 months. The participants with severe psychiatric or physical illness, body mass index (BMI) <18.5 kg/m^2^, and pregnant women were excluded from the study. The pilot and enrolment phase recruited further 15 006 participants, giving 16 138 in total. The overall response rate of those invited was equal to 93%. We included 14 916 subjects on the basis of inclusion criteria of the present study. Demographic information of participants was collected by questionnaire including age, gender, marital status, education level, etc. Blood samples were collected after an overnight fast of 12 hours. Fasting blood sugar (FBS), serum triglyceride (TG), high density lipoprotein (HDL) were determined by enzymatic method.

Moreover, height, weight, waist circumference and blood pressure of participants were measured. The method of measurements of mentioned factors has been explained in previously published article^9^ with more details. Women with waist circumference ≥88 cm and men with waist circumference of ≥102 cm were considered as abdominally obese.^10^ Subjects with SBP ≥130 and DBP ≥85 or antihypertensive drug treatment in a patient with a history of hypertension is an alternate indicator considered as patients with hypertension.^11^

### Study area

Azar cohort study is a part of a large Persian cohort study (The Prospective Epidemiological Research Studies of the Iranian Adults)^9,12^ which was launched at October 2014 and finished at January 2017. Azar cohort was established in Shabestar in East Azarbaijan province (North-west of Iran). It is conducted in 3 phases of pilot, enrollment and follow-up.

Shabestar is the capital of Shabestar city, East Azerbaijan province, Iran. At the 2011 census, its population was equal to 25 663, with 4824 families. It is located in proximity to Tabriz, the provincial capital, on the main Iranian-International railway line which connects Tehran and Tabriz to Turkey and Europe. Considering the proximity to Lake Urmia, the city experiences mild weather with wet winters, and summers are somewhat hot and dry during day time and cooler at night.

### Measures

*Socioeconomic status*

Socioeconomic status (SES) was defined based on job categories, education levels, and the family assets by using principal component analysis (PCA). SES classified into very high, high, middle, low and very low based on quintuple of obtained scores.

*Metabolic syndrome definition*

We defined metabolic syndrome according to the National Cholesterol Education Program’s Adult Treatment Panel III report (ATPIII) criteria.^13^ Subjects with 3 or more, the following conditions were defined as having the MetS: Waist circumference ≥102 cm in men and ≥88 cm in women, TG ≥150 mg/dL (drug treatment for elevated TGs is an alternate indicator), HDL-C <40 mg/dL in men and <50 mg/dL in women. Elevated blood pressure systolic ≥130 and/or diastolic ≥85 mm Hg (antihypertensive drug treatment in a patient with a history of hypertension is an alternate indicator). Elevated fasting glucose ≥100 (drug treatment of elevated glucose is an alternate indicator).

*Sleep habits*

Sleep habits of population were assessed using Pittsburgh questionnaire. Sleep habits information were presented in 3 forms: 24 hours sleep duration which was a sum of nocturnal and day time sleep hours, night sleep duration showed sleep duration at night and day time napping indicated sleep duration in day. Total sleep duration and night sleep duration was classified into 3 groups: <6 hours, 6-9 hours, and >9 hours. In addition, day time napping was categorized to: subjects, who were not napped, 0.25-2 hours, and >2 hours.

### Statistical analysis

Data were reported as mean, ± SD and also number (percent) where applicable. Comparisons were done using χ^2^ test for the categorical variables between groups (education level, marital status, etc). We used backward logistic regression analysis for estimating adjusted odds ratios (ORs) and their corresponding 95% CI. 6-9 hours’ sleep was considered as reference group in all analyses and those who not napped was considered as reference group in day time napping. Moreover, those who watched TV less than 2 hours and those who not have night shift work were determined as reference group. Data analysis was performed with SPSS Statistics for Windows version 21.0 (IBM Inc., Armonk, NY, USA) and the level of significance was set at 0.05.

## Results

As indicated in Table 1, among 14 916 participants, 5104 (34.22%; 95% CI: 33.44% to 34.96%) had MetS which was higher in women rather than men (41.0% vs. 25.8%). The prevalence of MetS among illiterates, those who divorced and those lived in rural area was significantly more than other groups (*P* < 0.001). Moreover, the prevalence of MetS in low and very low SES classification was significantly higher than other classifications (*P* < 0.001). Our findings indicated that MetS was more prevalent in those who used hypnotic drugs rather than those who did not use (*P* < 0.001). The prevalence of MetS in people who were in long sleep duration (>9 hours per night) and napping (>2 h/day) categories was higher than the other groups. In addition, we observed that a greater percentage of subjects who were watched TV over 2 hours had MetS compared to those who watched less than 2 h/day (36.9% vs 32.9; *P* < 0.001 ) (Table 1).

According to the results of backward logistic regression analysis, age, living place, BMI, hypnotic drug use, sleep and napping duration and TV time were the risk factors of MetS (Table 2). The odds of MetS in women was equal to 1.51 (95% CI: 1.39-1.67) times more than men (*P* < 0.001). Marriage and divorce in comparison to single, increase in risk of MetS respectively by 1.92 (1.27-2.19) and 2.32 (1.49-3.60) times. We observed that, the risk of MetS increased by increasing the age and BMI. Moreover, long sleep duration (>9 h/24 h), napping (0.25-2 h/day), hypnotic drug use and watching TV (2 h/day) increased the risk of MetS. In this regard, long sleep duration (>9 h/24 h), napping (0.25-2 h/day), hypnotic drug use and watching TV (2 h/day) increased the risk of MetS by 1.18 (1.05-1.33), 1.16 (1.07-1.26), 1.35 (1.13-1.60), and 1.13 (1.04-1.23) respectively.

## Discussion

Nowadays, prevalence of MetS parallel is increasing worldwide by increase in obesity. So, it can be considered as a silent killer. Moreover, sleep disturbance becomes a common problem in recent years.^14^ On the other hand, it has been known that sleep duration plays an important role in maintaining a good health.^5^ In this regard, U shaped association has been reported between sleep duration and increased risk of mortality.^15,16^

The results of this cross-sectional study with large sample size showed that long sleep duration were significantly increased the risk of MetS which is consistence with Jike et al’s study. They reported that sleep more than 8-9 h/day are significantly associated with cardiovascular disease and mortality.^17^ The findings of another study indicated that short and long sleep duration increased the risk of metabolic syndrome.^8^ In contrast to our results, previous studies reported that there is a positive correlation between short sleep duration (<6 h) and MetS components.^7,18,19^ the discrepancy between our results and other studies may be due to the self-report sleep and different definitions of short or long sleep duration.

Also, we found that, day time napping increased the risk of MetS. In line with these results, Guo et al observed that day time napping over 1 hour increased the risk of type II diabetes.^20^ In a cohort study on retired worker indicated that long afternoon napping can be considered as a novel risk factor of Type II diabetes. In another population based study, Lin et al reported that napping increased the risk of MetS in non-obese Chinese women.^21^ Yang et al found that long duration of napping was positively associated with MetS incidence.^22^

By these mechanisms, long duration of napping which increased the risk of MetS has not been fully understood. On the basis of previous studies, some mechanisms have been proposed for this association. Long duration napping reduced energy expenditure which is linked with obesity and its consequences. Moreover, it has been reported that disrupting of circadian rhythm involved in insulin resistance and diabetes mellitus. On the other hand, evening cortisol concentration elevated in the nap takers, that lead to subjects become predisposed to insulin resistance.

After adjusting the confounder factors, those who watched TV more than 2 hours had 13% (OR 1.13; 1.04-1.23) more risk of MetS compared to those watched ≤2 hours. Of course, it should be noted the observed significance in narrow OR may be due to large sample size. These results are in line with previous studies which conducted on leisure time and cardiometabolic risk factors. Rosique-Esteban et al observed that long watching TV increased the risk of obesity, type II diabetes and metabolic syndrome components.^23^ Results of another study indicated that less physical activity and more watching TV are significantly associated with MetS.^24^

In the present study, those who used hypnotic drugs had 35% (OR: 1.35; 1.13-1.60) more risk for metabolic syndrome compared to those who did not use. This finding is supported by previous study which showed there is a positive correlation between hypnotic drug use and MetS.^25^ The precise mechanism of how hypnotic drug leads to increase in the risk of MetS is not known. It has been assumed that these drugs change life style by reducing physical activity.^26^ Moreover, most subjects who use hypnotic drugs have other psychiatric problems such as depression^27^ which may increase the risk of MetS.^24^

The results of the present study should be interpreted considering the following limitations: (1) the cross-sectional design of the study, which implies that no causal inferences could be made. (2) Using subjective measurements of sleep pattern that may be a potential for misreporting. The strength of this study is the assessment of sleep habits and MetS components in large population.

## Conclusion

The findings of present study showed that long sleep duration and napping in 24 hours increased the risk of MetS. Moreover, we found that watching TV more than 2 h/day is significantly associated with MetS. According to these results, it appears that proper education for improvement of sleep habit is necessary to reduce incidence of MetS and its consequences. However, for more precise results, there is need to more longitudinal researches and using objective method of sleep habits evaluation.

## Ethical approval

This study was approved by Ethics Committee of Tabriz University of medical sciences (TBZMED.REC.1393.205). After a full explanation of the study was provided, and prior to commencing participation, all participants provided written informed consent.

## Competing interests

The authors declared no potential conflicts of interest with respect to the research, authorship, and/or publication of this article.

## Funding

Liver and gastrointestinal diseases research center, Tabriz University of Medical Sciences.

## Authors’ contributions

Conception and design: MHS. Doing the field, experimental, clinical, data collection or compilation work, provided there is also a scholarly input during the process: EF. Data analysis and interpretation: EF, ZN. Preparation of draft manuscript, doing revisions or providing critique: ZN, EF, ARO. Overall and/or sectional scientific management: MHS.

## Acknowledgments

The authors are grateful for the financial support of the liver and gastrointestinal disease research center, Tabriz University of Medical Sciences. The authors also are deeply indebted to all subjects who participated in this study. We appreciate the contribution by the investigators and the staff of the Azar cohort study. We thank the close collaboration of the Shabestar health center. In addition, we would like to thank the Persian cohort study staff for their technical support.

**Table 1:** Baseline characteristics of subjects stratified by metabolic syndrome

	**Metabolic syndrome**						***P*** value*
	**No**		**Yes**		**Total**		
	**No.**	**%**	**No.**	**%**	**No.**	**%**	
Gender							
Men	4951	74.2	1724	25.8	6675	44.8	<0.001
Women	4861	59	3380	41	8241	55.2	
Total	9812	65.8	5104	34.2	14916	100	
Education level							
Illiterate	1315	51.8	1226	48.2	2541	16.85	<0.001
Elementary	4778	65.1	2560	34.9	7338	49.2	
Middle school	1472	72	573	28	2045	13.71	
Diploma	1259	74.3	436	25.7	1695	11.36	
College	988	76.3	307	23.7	1295	8.68	
Marital status							
Single	199	85.8	33	14.2	232	1.55	<0.001
Married	9194	66.5	4632	33.5	13826	92.69	
Divorced	416	48.9	434	51.1	850	5.69	
Age groups (y)						
35-39	2009	80.5	487	9.5	2496	16.7	<0.001
40-44	1984	75.1	658	12.9	2642	17.7	
45-49	1813	67.8	860	16.8	2673	17.9	
50-54	1491	61.2	944	38.8	2435	16.3	
55-59	1128	54.7	936	45.3	2064	13.8	
60-64	832	54.8	685	45.2	1517	10.2	
65-72	555	51.0	534	49	1089	7.3	
BMI (kg/m^2^) classification						
18.5-24.9	2861	92.4	234	7.6	3095	20.9	<0.001
25-29.9	4347	71.0	1772	29.0	6119	41.3	
≥30	2513	44.8	3098	55.2	5611	37.8	
Socioeconomic status						
Very high	1991	70.2	847	29.8	2838	19.1	<0.001
high	1937	67.7	925	32.3	2862	19.3	
Middle	1925	66.3	980	33.7	2905	19.6	
low	1903	63.8	1081	36.2	2984	20.1	
Very low	2011	62.0	1231	38	3242	21.9	
Living place							
Urban	6934	66.8	3444	33.2	10378	70	<0.001
rural	2878	63.4	1660	36.6	4538	30	
Hypnotic drug use							
Yes	399	53.5	347	46.5	746	5	<0.001
no	9413	66.4	4757	33.6	14170	95	
Total Sleep duration, h/day						
6-9	7733	67.0	3809	33.2	11542	77.37	<0.001
<6	994	63.0	583	37.3	1577	10.5	
>9	1085	60.0	712	39.8	1797	12.04	
Nocturnal sleep duration, h/day						
6-9	7051	66.8	3511	33.2	10562	70.8	<0.001
<6	2358	64.4	1306	35.6	3664	24.6	
>9	403	58.4	287	41.6	690	4.62	
TV time, h/day						
≤2	6793	67.1	3337	32.9	10130	44.40	<0.001
>2	3019	63.1	1767	36.9	4786	55.58	
Napping duration, h/day						
0	4821	68.3	2236	31.7	7057	47.05	<0.001
0.25-2	4884	63.6	2793	36.4	7677	51.18	
>2	107	58.8	75	41.2	182	1.7	
Shift work at night						
Yes	916	74.6	312	25.4	1228	8.23	<0.001
No	8896	65	4792	35	13688	91.8	
Shift work at night (day/year)						
0	8895	65.0	4792	35	13687	91.2	<0.001
1-24	688	75.5	223	24.5	911	6.0	
≥24	229	72	89	28	318	2.1	

* *P* value: χ^2^ test.

**Table 2:** Odds ratios (with 95% CIs) of metabolic syndrome on the basis of sleep habits

**Variables**	**Metabolic syndrome**		***P*** value*
	**OR**	**95% CI**	
Gender			
Men	Reference		
Women	1.51	1.39-1.64	<0.001
Marital status			
Single	Reference		
Married	1.92	1.27-2.19	0.002
Divorced	2.32	1.49-3.60	<0.001
Age groups (y)		
35-39	Reference		
40-44	1.22	1.06-1.41	0.004
45-49	1.68	1.46-1.93	<0.001
50-54	2.41	2.1-2.77	<0.001
55-59	3.30	2.86-3.81	<0.001
60-64	3.51	3.00-4.11	<0.001
65-72	4.58	3.84-5.47	<0.001
Living place		
Urban	Reference		
Rural	1.10	1.01-1.20	0.01
BMI (kg/m^2^) classification		
18.5-24.9	Reference		
25-29.9	4.99	4.30-5.78	<0.001
≥30	14.48	12.48-16.80	<0.001
Hypnotic drug use		
No	Reference		
Yes	1.35	1.13-1.60	0.001
Total Sleep duration, h/day		
6-9	Reference		
<6	0.97	0.85-1.10	0.64
>9	1.18	1.05-1.33	0.006
Napping duration, h/day		
0	Reference		
0.25-2	1.16	1.07-1.26	<0.001
>2	1.16	0.83-1.64	0.37
TV time, h/day		
≤2	Reference		
>2	1.13	1.04-1.23	0.002

*P value: backward logistic regression.
